# Psychopathological symptoms, personality, and epistemic stances in individuals with myocardial infarction: an empirical investigation

**DOI:** 10.3389/fpsyg.2025.1587747

**Published:** 2025-05-22

**Authors:** Gianluca Cruciani, Marianna Liotti, Annalisa Tanzilli, Gabriele Lo Buglio, Davide Guarino, Nicola Carone, Matteo Casali, Federica Galli, Vittorio Lingiardi

**Affiliations:** ^1^Department of Systems Medicine, University of Rome Tor Vergata, Rome, Italy; ^2^Department of Dynamic and Clinical Psychology, and Health Studies, Faculty of Medicine and Psychology, Sapienza University of Rome, Rome, Italy; ^3^Department of Biology and Biotechnologies “Charles Darwin”, Sapienza University of Rome, Rome, Italy

**Keywords:** myocardial infarction, psychopathology, anxiety, depression, personality, epistemic trust, epistemic mistrust

## Abstract

**Introduction:**

Myocardial infarction (MI) is a major cause of mortality worldwide. Psychopathological symptoms play a bidirectional role in MI prognosis, both increasing cardiovascular risk and being exacerbated by cardiac events, leading to further complications. Personality impairments and disruptions in epistemic trust—the ability to assess social communications as trustworthy and relevant—strongly affect psychopathology levels and may worsen MI clinical outcomes by impeding health behaviors and treatment adherence. This is the first study examining the interplay between psychopathological symptoms, personality dysfunction, and epistemic dimensions in MI patients compared to healthy controls.

**Methods:**

A sample of 67 MI patients and 80 age- and gender-matched healthy controls completed self-report measures to assess levels of general psychopathology (DSM−5 self-rated Level 1 Cross-Cutting Symptom Measure), personality functioning (Personality Inventory for DSM-5 Short Form), and epistemic stances (Epistemic Trust, Mistrust, and Credulity Questionnaire). Multivariate analyses were used to compare the groups, while correlations and moderation models were employed to evaluate associations among variables within the MI group.

**Results:**

MI patients showed significantly higher psychopathological symptoms, more severe personality impairments, and greater epistemic mistrust than controls. Within the MI group, psychopathological symptoms were associated with specific maladaptive personality traits (especially, negative affectivity) and epistemic mistrust and credulity. The relationship between worse personality functioning and severe psychopathological symptoms was moderated by epistemic mistrust.

**Discussion:**

The study emphasizes the importance of addressing psychopathology and epistemic disruptions in clinical settings to improve the treatment's adherence and recovery. The development of targeted interventions to mitigate psychological vulnerabilities in MI patients is recommended.

## 1 Introduction

Cardiovascular diseases (CVDs) remain the leading cause of death worldwide, with a rising incidence and prevalence over the past four decades (Mohebi et al., 2022; Townsend et al., 2022; Zhao, 2021). Among these, acute coronary syndromes (ACSs), in particular myocardial infarction (MI), contribute significantly to the alarming global mortality rates (Salari et al., 2023). MI results from a critical reduction or blockage of blood flow in a coronary artery, leading to tissue necrosis, and can be further clinically classified into ST-segment elevation MI (STEMI) and non-ST-segment elevation MI (NSTEMI) (Thygesen et al., 2019). A major concern in MI survivors is recurrence, with 11–14% of patients experiencing rehospitalization within 30 days and up to 49% within 1 year (Khot et al., 2018; Wang et al., 2019). Both unmodifiable (e.g., male gender, older age) and modifiable factors (e.g., diabetes, hypertension, unemployment, non-adherence to treatment) contribute to this risk (Arnold et al., 2015; Kim et al., 2018; Kwok et al., 2020).

Mental health problems play a crucial role in cardiovascular outcomes, forming a bidirectional relationship with CVDs: on one hand, various forms of mental suffering (e.g., depression, anxiety, other mood disorders, schizophrenia/psychotic disorders, and post-traumatic stress) can increase cardiovascular risk (Sreenivasan et al., 2021); on the other, CVDs themselves can trigger psychological distress, which consequently raises the likelihood of recurrent adverse events (Goldstein et al., 2015; Sreenivasan et al., 2022). Notably, rehospitalization rate is estimated to be 5% greater for MI patients with past-year psychiatric comorbidity (Ahmedani et al., 2015), comparable to rehospitalizations due to physiological conditions (Andrés et al., 2012). Considering the role of mental distress among patients with MI, it becomes essential to explore their psycho(patho)logical functioning which may shape their emotional and behavioral responses to several conditions.

Personality emerges as a critical factor, influencing coping mechanisms, interpersonal patterns, and overall mental capacities (Barańczuk, 2019; Connor-Smith and Flachsbart, 2007). By determining patients' emotional processing, social interactions, and stress responses, personality may significantly impact post-MI health and long-term prognosis. The association between personality and CVDs has been extensively studied. Early research identified higher risks for cardiac events in individuals displaying aggressiveness, hostility, and competitiveness, traits commonly labeled as Type A personality (Friedman and Rosenman, 1959; Rosenman and Friedman, 1959). Later, a Type D personality profile—marked by negative affectivity and social inhibition—was also linked to poorer cardiovascular outcomes and increased mortality risk (Denollet et al., 1995; Grande et al., 2012). More recently, studies on dimensional models of personality, such as the Big Five, suggested that higher levels of neuroticism and extraversion were associated with greater risk of adverse cardiac events, while greater conscientiousness and openness had protective effects (Agvall and Jonasson, 2025; Dahlén et al., 2022; Jokela et al., 2014; Lee et al., 2014; Otonari et al., 2021).

While the relationship between personality and cardiovascular health is well-established, the underlying mechanisms through which personality affects health-related behaviors and treatment adherence remain insufficiently explored. In this regard, the emerging theoretical framework of epistemic trust (ET) offers a promising avenue. ET refers to an individual's capacity to effectively evaluate social communications as trustworthy, relevant, and generalizable to broader contexts (Fonagy et al., 2015). This construct has been increasingly recognized as a key dimension of human social learning and adaptation, shaping how individuals engage with and internalize interpersonally transmitted knowledge (Fonagy and Allison, 2014; Campbell et al., 2021). ET plays a fundamental role in psychological and personality functioning, allowing individuals to flexibly integrate new information and adjust in response to changes (Luyten et al., 2020). Conversely, disruptions in ET—often associated with trauma—can result in excessive skepticism (i.e., epistemic mistrust, EM) or uncritical acceptance (i.e., epistemic credulity, EC) toward information conveyed by others and have been associated (particularly for what it concerns EM) with a range of psychological vulnerabilities, including mental health symptoms, deficit in mentalization, emotional dysregulation, maladaptive stress responses, and interpersonal problems (Campbell et al., 2021; Fiorini Bincoletto et al., 2025; Fonagy and Campbell, 2017; Liotti et al., 2023; Talia et al., 2019; Tanzilli et al., 2022). Since disruptions in ET may contribute to heightened stress reactivity and maladaptive coping mechanisms as well as to impairments in psychological and personality functioning, they may increase the risk of CVDs. Furthermore, given that MI itself constitutes a psychologically distressing, if not traumatic, event, it may exacerbate pre-existing vulnerabilities in ET, further complicating recovery trajectories. Indeed, following a cardiovascular event, the ability to accurately trust medical guidance is paramount for optimizing recovery. MI patients need to engage in a collaborative relationship with healthcare providers, adhere to complex treatment regimens, and make significant lifestyle changes (Levine et al., 2021); however, individuals with disruptions in ET (particularly mistrustful ones) may struggle to incorporate medical recommendations, leading to poorer adherence to treatment and an increased risk of adverse outcomes. ET is thus crucial for MI patients, as it may directly influence their ability to engage with healthcare providers, adhere to prescribed medications, and adopt essential lifestyle changes, such as smoking cessation, dietary modifications, regular physical activity, and stress management—all of which are critical for secondary prevention and long-term recovery. Moreover, while EM may stem from pre-existing personal experiences, it is possible that it can also be enhanced by the MI event itself, particularly if patients experience heightened distress, perceive medical information as inconsistent, or feel dismissed or unsupported in their interactions with healthcare providers (cf. Fisher et al., 2025). A strained patient-provider relationship, characterized by poor communication or lack of perceived empathy, may further reinforce EM, leading patients to disengage from medical recommendations and adopt maladaptive coping strategies. In this perspective, understanding how personality and ET interact to shape cardiovascular risk and recovery may have crucial implications for clinical practice, providing novel insights into how to identify at-risk individuals before and after CVDs, as well as to develop effective interventions in the pursuit of holistic, tailored, and patient-centered care.

In line with what has been reported so far, the aim of the current study was threefold:

1) To explore differences in general psychopathological symptomatology, personality traits and epistemic stances between people who had experienced MI and healthy controls. Based on previous research (Dahlén et al., 2022; Kupper and Denollet, 2018), it was hypothesized that MI patients would display higher levels of psychological symptoms and more dysfunctional personality patterns than healthy controls. Despite the lack of empirical literature in this field, MI patients would also show greater ET disruptions than controls—particularly regarding EM—given its well-documented association with psychopathological variables (Campbell et al., 2021; Li et al., 2022).
2) To explore associations between psychopathological symptoms, personality functioning and epistemic stances within the MI group. According to studies conducted in other populations (Campbell et al., 2021; Fiorini Bincoletto et al., 2025; Liotti et al., 2023; Tanzilli et al., 2022), higher symptom severity would be correlated with personality dysfunction and disruptions in ET (i.e., higher levels of EC and, particularly, EM).
3) To assess whether epistemic stances moderate the relationship between personality functioning and psychopathological symptoms (Andersen and Bienvenu, 2011; Widiger et al., 2019) in MI patients. Specifically, EM would modulate this association (cf., Fiorini Bincoletto et al., 2025; Li et al., 2022).

## 2 Materials and methods

### 2.1 Participants

Sixty-seven patients (mean age = 61.60 ± 9.77 years) admitted to the Department of Clinical Internal, Anaesthesiologic, and Cardiovascular Sciences of the Sapienza University of Rome for a MI episode were recruited as clinical group. The diagnosis of acute MI has been made following the guidelines of the European Society of Cardiology for managing acute MI in patients presenting NSTEMI and STEMI (Collet et al., 2021; Ibanez et al., 2018). Participants were excluded if they were below 18 years of age, not fluent in Italian, or were unable to understand the research's instructions. Participants were also excluded if they had active cancer, liver cirrhosis, chronic infectious or autoimmune disease, as these are conditions that were shown to potentially affect patients' emotional and cognitive responses (e.g., Agidigbi et al., 2025; Baziliansky and Cohen, 2021; Carrozzino and Porcelli, 2018). All inclusion and exclusion criteria were assessed through a preliminary unstructured interview to check for participant's eligibility.

The control group was composed of 80 gender- and age-matched healthy participants (mean age = 59.00 ± 8.46 years; gender distribution comparison with clinical group: χ^2^ = 0.985; *p* = 0.321; mean age comparison: *t*_(145)_ = 1.728; *p* = 0.086). Healthy participants were recruited from the general population by word of mouth and using advertising shared on social media. Control participants were excluded if they had previous or current CVDs, were below 18 years of age, not fluent in Italian, had active cancer, liver cirrhosis, chronic infectious or autoimmune disease, were unable to understand the study's instructions, were active smokers or had a Body Mass Index (BMI) ≥30. Healthy participants were recruited over a 3-month period following the data collection from MI patients to ensure matching based on age and gender. Eligibility was assessed through an initial screening in which potential participants were required to confirm whether they met the inclusion and exclusion criteria. Recruitment was carried out via social media, specifically Facebook and Instagram, as well as through word of mouth, which involved direct outreach within community networks, including personal and professional contacts.

### 2.2 Procedure

The present study was approved by the ethics committee of the Department of Dynamic and Clinical Psychology, and Health Studies, Sapienza University of Rome (Protocol Number 0000148/2022 of 04/02/2022), and conforms to the World Medical Association Declaration of Helsinki of 1975, as revised in 2008. All participants participated voluntarily in the study, provided written informed consent before the experimental procedure, and were free to withdraw from the study at any time without any consequences.

Data were collected through an online survey with SurveyMonkey including socio-demographic characteristics, clinical information, and three self-reported questionnaires (see “Measures” section below). Data collection granted participants anonymity by employing an alphanumeric code; it lasted ~45 min. MI patients completed the research protocol before hospital discharge; particularly, assessments were conducted in person while MI patients were still hospitalized, and the data collection process was supervised by trained researchers who were present to provide clarification on procedural aspects and to assist patients if needed. However, researchers ensured a neutral stance throughout the assessment, strictly avoiding any form of guidance or influence on participants' responses to maintain the integrity of the self-report measures.

### 2.3 Measures

#### 2.3.1 Sociodemographic and clinical information

An *ad hoc* questionnaire was developed to collect data regarding sociodemographic (i.e., gender, age, marital status, education) and clinical (i.e., BMI, smoking habits, alcohol consumption, previous heart diseases, dyslipidaemia, insulinemia, pulmonary oedema, transient ischemic attacks, renal failure, diabetes, chronic obstructive pulmonary disease, sleep apnea, neoplasms) information.

#### 2.3.2 Psychopathological symptoms

General psychopathological symptomatology was measured using the DSM−5 self-rated Level 1 Cross-Cutting Symptom Measure (DSM-5-CCSM—Narrow et al., 2013; American Psychiatric Association, 2013), a 23 items self-report questionnaire which assesses 13 relevant mental health domains (i.e., depression, anger, mania, anxiety, somatic symptoms, suicidal ideation, psychosis, sleep problems, memory, repetitive thoughts and behaviors, dissociation, personality functioning, and substance use). Items evaluated how much or how often the person has been affected by a specific symptom over the previous 2 weeks on a 5-point Likert scale from 0 (“none, not at all”) to 4 (“severe, nearly every day”), with higher scores indicating greater symptom severity. It is possible to calculate a total score indicating general psychopathological symptomatology derived by the mean of the single domains' scores, which was used in the present study. The scale showed excellent reliability, with Cronbach's alpha value of 0.96.

#### 2.3.3 Personality traits

Personality was assessed using the Personality Inventory for DSM-5 Short Form (PID-5; Maples et al., 2015; Somma et al., 2019), a 100-item self-report measure of the DSM-5 alternative personality disorder model traits which provides a score for each of the following 5 personality domains: negative affectivity, detachment, antagonism, disinhibition, and psychoticism. Items are assessed on a Likert scale ranging from 0 (“very false or often false”) to 3 (“very true or often true”). Higher scores in each domain indicate greater dysfunction in the corresponding personality trait. An overall index (i.e., total score) of personality dysfunction was also calculated by dividing the total raw score by the total number of items in the measure. The PID-5 domains align with the Five Factor Model (FFM), such that negative affectivity converges into FFM neuroticism, detachment into FFM introversion, psychoticism into low FFM openness, antagonism into low FFM agreeableness, and disinhibition into low FFM conscientiousness (Costa and McCrae, 1995; Fowler et al., 2015; García et al., 2022). The scale showed good reliability, with Cronbach's alpha values of 0.82, 0.84, 0.77, 0.79, and 0.82 for negative affectivity, detachment, antagonism, disinhibition, and psychoticism scales, respectively.

#### 2.3.4 Epistemic trust, mistrust, and credulity

Epistemic stances were assessed using the Epistemic Trust, Mistrust, and Credulity Questionnaire (ETMCQ—Campbell et al., 2021; Liotti et al., 2023), a 15-item self-report questionnaire measuring epistemic trust (ET), mistrust (EM), and credulity (EC). Items are assessed on a Likert scale ranging from 1 (“strongly disagree”) to 7 (“strongly agree”). The scale showed good reliability, with Cronbach's alpha values of 0.73, 0.82, and 0.74 for ET, EM, and EC scales, respectively.

### 2.4 Statistical analyses

All analyses were run using JAMOVI version 2.4.11, and the jAMM statistical package (Gallucci, 2021). Statistical significance was set at *p* < 0.05. Preliminary group differences in sociodemographic and clinical information were first run to provide a general sample description.

Two univariate analyses of variance (ANOVAs) were performed to assess group differences between MI patients and healthy controls in overall psychopathological symptomatology and global personality functioning, with the DSM-5-CCSM and the PID-5 total scores as dependent variables, respectively. Two multivariate analyses of variance (MANOVAs) were also run to evaluate group differences in personality traits and epistemic stances: the PID-5 domains (i.e., negative affectivity, detachment, antagonism, disinhibition, and psychoticism) were used as dependent variables in the first MANOVA, while the three ETMCQ subscales (i.e., ET, EM, and EC) were dependent variables in the second MANOVA. Effect sizes were calculated using partial eta squared (η*p*^2^). To explore possible associations between general psychopathological symptomatology, personality traits, and epistemic stances among MI patients, bivariate correlations (Pearson's *r*, 2-tailed) were run between the DSM-5-CCSM total score, the PID-5 domains and the ETMCQ subscales. Magnitude of correlations was estimated considering Cohen's guidelines (cf. Cohen, 1988).

Additionally, to assess whether epistemic stances could moderate the association between personality functioning and psychopathological symptoms in MI patients, a General Linear Model (GLM) was run considering the PID-5 overall index of personality functioning, the EM scale and their interaction as independent variables, and the DSM-5-CCSM total score as dependent variable. Simple effects were specifically carried out to address the possible moderator role of the EM in the association between the PID-5 personality overall index and the DSM-5-CCSM score. Among epistemic stances, the choice to include in the GLM only the EM was theory-driven, as it was previously proposed that mistrust may be considered a more significant risk factor for the development of psychological symptoms than other epistemic stances (Benzi et al., 2023; Fiorini Bincoletto et al., 2025). Cohen's ƒ^2^ was used to evaluate the effect size for the GLM (cf. Cohen, 1988).

## 3 Results

Descriptives and group differences in sociodemographic and clinical information are fully reported in Supplementary Table S1. In contrast, a comprehensive picture of group differences with descriptives on the study's psycho(patho)logical variables was reported in Table 1.

**Table 1:** Group differences with descriptives of MI patients and healthy controls' scores in PID-5, ETMCQ and DSM-5-CCSM.

**Measure**	**MI patients mean ±SD**	**Healthy controls mean ±SD**	***F***	***p***	***ηp* ^2^**
DSM-5-CCSM	44.72 ± 14.05	12.06 ± 12.15	228.402	<0.001	0.612
**PID-5**					
Negative affectivity	1.49 ± 0.80	0.90 ± 0.64	24.476	<0.001	0.144
Detachment	0.03 ± 0.75	0.67 ± 0.61	5.539	0.020	0.037
Antagonism	0.51 ± 0.49	0.45 ± 0.39	0.714	0.400	0.005
Disinhibition	1.04 ± 0.76	0.61 ± 0.44	6.874	<0.001	0.115
Psychoticism	0.70 ± 0.61	0.42 ± 0.42	10.692	0.001	0.069
Total score	0.93 ± 0.54	0.61 ± 0.42	16.873	<0.001	0.104
**ETMCQ**					
Trust	4.09 ± 1.00	4.35 ± 1.16	1.950	0.165	0.013
Mistrust	4.52 ± 1.56	3.45 ± 1.08	24.161	<0.001	0.143
Credulity	2.64 ± 1.36	2.46 ± 1.26	0.750	0.388	0.005

DSM-5-CCSM, DSM−5 self-rated Level 1 Cross-Cutting Symptom Measure; PID-5, Personality Inventory for DSM-5 Short Form; ETMCQ, Epistemic Trust, Mistrust and Credulity Questionnaire.

The results derived from the first ANOVA, including Group as the independent variable and the DSM-5-CCSM total score as the dependent variable, showed more severe psychopathological symptoms among MI patients than healthy controls. Similarly, the second ANOVA with the PID-5 global personality functioning as the dependent variable revealed that MI patients were significantly more impaired than healthy controls. Regarding group differences in personality traits, the MANOVA including PID-5 domains as dependent variables yielded significant results: MI patients showed higher scores of negative affectivity, detachment, disinhibition, and psychoticism than healthy controls, indicating greater dysfunctions in these personality traits. Concerning epistemic stances, findings from the second MANOVA revealed that MI patients had significantly higher scores in EM than the healthy group.

Bivariate correlations between DSM-5-CCSM total score, PID-5 domains, and global index, and ETMCQ subscales among the MI group are fully reported in Table 2. Results showed that MI patients' psychopathological symptoms have significant positive correlations with all personality traits and the level of overall personality impairment, as well as with EM and EC (cf. Cohen, 1988).

**Table 2:** Correlations between PID-5 domains (i.e., negative affectivity, detachment, psychoticism, antagonism, and disinhibition) and PID-5 total score, ETMCQ subscales (i.e., trust, mistrust, and credulity) and DSM-5-CCSM total score among the MI group.

	**1**	**2**	**3**	**4**	**5**	**6**	**7**	**8**	**9**	**10**
1. DSM-5-CCSM	1									
**PID-5**									
2. Negative affectivity	0.61^***^	1								
3. Detachment	0.47^***^	0.63^***^	1							
4. Antagonism	0.50^***^	0.47^***^	0.25^*^	1						
5. Disinhibition	0.66^***^	0.57^***^	0.46^***^	0.65^***^	1					
6. Psychoticism	0.62^***^	0.49^***^	0.45^***^	0.66^***^	0.77^***^	1				
7. Total score	0.73^***^	0.82^***^	0.74^***^	0.71^***^	0.86^***^	0.83^***^	1			
**ETMCQ**									
8. Trust	−0.10	−0.12	−0.13	0.05	−0.06	−0.02	−0.08	1		
9. Mistrust	0.67^***^	0.44^***^	0.27^*^	0.59^***^	0.56^***^	0.54^***^	0.59^***^	−0.10	1	
10. Credulity	0.30^*^	−0.30^*^	0.04	0.17	0.28^*^	0.24	0.26^*^	0.08	0.17	1

DSM-5-CCSM, DSM−5 self-rated Level 1 Cross-Cutting Symptom Measure; PID-5, Personality Inventory for DSM-5 Short Form; ETMCQ, Epistemic Trust, Mistrust and Credulity Questionnaire.

^*^p ≤ 0.05, ^**^p ≤ 0.01, ^***^p ≤ 0.001.

**Figure 1:**
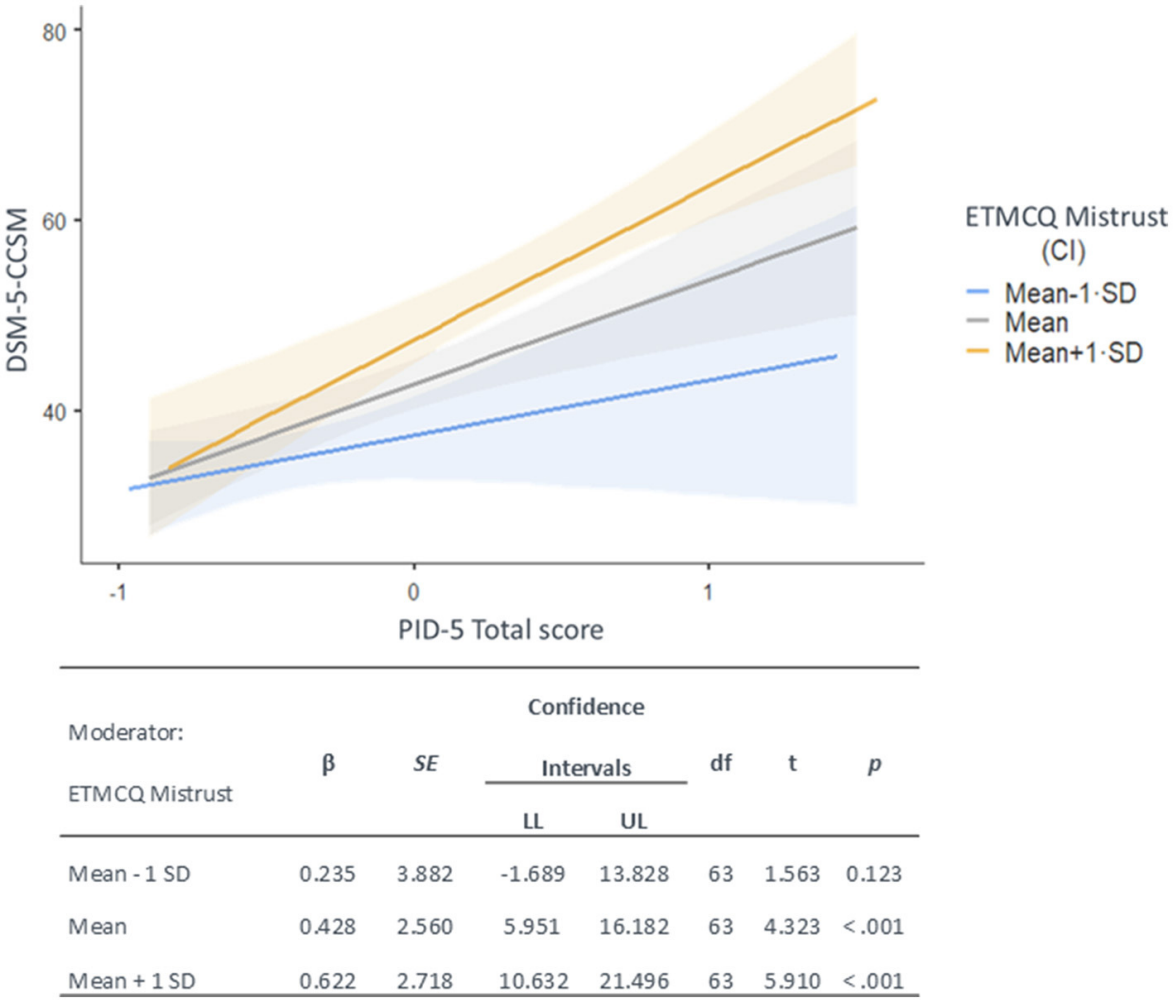
Interaction between the PID-5 total score of personality dysfunction and the ETMCQ mistrust scale on the DSM-5-CCSM total score, with simple effects.

The GLM with the PID-5 global index, EM scale, and their interaction as independent variables, and the DSM-5-CCSM total score as dependent variable yielded significant results, explaining 62.8% of the observed variance (Table 3). ƒ^2^ value was 0.27 reflecting a medium-large effect size. The GLM showed a significant effect of PID-5 overall index of personality impairment and EM on the DSM-5-CCSM total score, indicating that poorer psychopathological symptomatology was associated with higher dysfunctional personality traits and EM stance. Additionally, the GLM showed a significant interaction between the PID-5 global personality index and EM scale; simple effects showed that the degree of personality impairment has a significant effect on symptom severity when levels of EM were higher (Figure 1).

**Table 3:** GLM fit indices of variables associated with psychopathological symptomatology.

**Outcome: DSM-5-CCSM**		**β**	**SE**	**Confidence Intervals**		**d*f***	***t***	***p***
				**LL**	**UL**			
Intercept		−0.112	1.250	40.643	45.638	63	34.514	<0.001
PID-5 total score		0.428	2.560	5.951	16.182	63	4.323	<0.001
ETMCQ mistrust		0.427	0.865	2.108	5.565	63	4.435	<0.001
PID-5 total score ^*^ ETMCQ mistrust		0.193	1.384	0.432	5.962	63	2.310	0.024
*R*^2^ (explained variance)	0.628							

DSM-5-CCSM, DSM−5 self-rated Level 1 Cross-Cutting Symptom Measure; PID-5, Personality Inventory for DSM-5 Short Form; ETMCQ, Epistemic Trust, Mistrust and Credulity Questionnaire; SE, standardized error; df, degrees of freedom; LL, lower limit; UL, upper limit.

## 4 Discussion

Our findings sought to provide new insights into the interplay between psychological distress, maladaptive personality traits, and epistemic stances in patients who have experienced MI. Overall, this study highlights potential mechanisms linking these factors to an increased risk of myocardial infarction and impaired post-MI recovery, shedding light on the broader psychopathological and personological dynamics of patients, particularly their difficulties in building relationships rooted in trust and security.

Regarding the **first** study's aim, in line with the hypotheses, MI patients showed significantly higher psychopathological levels compared to controls. These findings are consistent with broader evidence linking psycho(patho)logical functioning to both cardiovascular vulnerability and poor recovery outcomes. MI patients exhibited significantly higher levels of negative affectivity, detachment, disinhibition, and psychoticism traits than controls, supporting the strong associations between maladaptive personality traits and CVD risk. While anxiety, depression, and other mental diseases are widely recognized as critical variables for CVDs morbidity and mortality, personality dimensions have been often overlooked in clinical settings. Personality has a relevant influence on stress responses and biological processes underlying cardiovascular health, including heart rate variability (Carpeggiani et al., 2005; Cruciani et al., 2023; Eikeseth et al., 2020; Lazzeroni et al., 2022; Zohar et al., 2013), cortisol levels (Oswald et al., 2006; Sundin et al., 2021), and inflammatory biomarkers (Allen and Laborde, 2017; Armon et al., 2013). Maladaptive personality traits also increase the likelihood of engaging in harmful activities, such as smoking, excessive alcohol consumption, and substance abuse (Yousef et al., 2024; Hall et al., 2014), worsening MI prognosis. For instance, it was previously found that high psychoticism traits, characterized by cognitive-perceptual dysregulation and detachment from reality, may be more pronounced in individuals with severe medical conditions (e.g., Ohseto et al., 2018). In patients with cardiovascular diseases, these traits could contribute to maladaptive interpretations of their condition, increased socio-emotional dysregulation, difficulties in engaging with medical recommendations and in adopting healthy behaviors (Narita et al., 2020; Stürmer et al., 2006).

Personality can also shape social and emotional adaptation post-MI. Traits such as elevated negative affectivity, disinhibition, and psychoticism—strongly linked to a higher risk of psychopathology (Gioletti and Bornstein, 2024; Mullins-Sweatt et al., 2019; Pollock et al., 2016)—may amplify patients' vulnerability after a cardiovascular event. Negative affectivity may trap individuals in a cycle of intense anxiety, sadness, or anger, diminishing their emotional regulation, coping abilities, and overall resilience. Disinhibition is associated with impulsive behaviors and poor decision-making abilities; in this population, it could be translated into risky behaviors and reduced adherence to therapeutic recommendations. Psychoticism, marked by distorted thinking and maladaptive interpretations of reality, may lead MI patients to dysfunctional interpretations of their condition or others' intentions (including healthcare providers), hindering effective help-seeking, use of social support, and recovery (e.g., Tanzilli et al., 2022). Furthermore, all these personality traits, in particular detachment, can intensify a sense of isolation and loneliness, a significant risk factor for poor cardiovascular outcomes (Freilich et al., 2024; Hodgson et al., 2020).

Although interpersonal relationships and social learning mechanisms play a crucial role in health behaviors and recovery, these dimensions are scarcely explored among CVDs. Our results show that MI patients displayed significantly higher levels of EM than healthy controls, suggesting that a pervasive tendency to doubt, distrust, and dismiss socially transmitted information might be an intrinsic feature of their psychological profile. EM is associated with both maladaptive personality functioning and psychopathological symptoms (Campbell et al., 2021; Liotti et al., 2023; Li et al., 2022), indicating that mistrustful stances may amplify psychological distress and maladaptive coping strategies in individuals with pre-existing vulnerabilities, thereby exacerbating the CVDs' clinical conditions.

Building on these findings, it was examined how psychopathology, personality traits, and epistemic stances were interconnected within the MI sample. Significant correlations emerged between maladaptive personality traits and psychopathological symptoms, reinforcing the notion that personality shapes psychological distress post-MI, dynamically interacting with contextual factors. The relationship between personality traits and clinical symptoms is widely recognized as multidirectional (Andersen and Bienvenu, 2011; Widiger et al., 2019). Maladaptive traits may intensify the overall psychological burden in this population, shaping how symptoms such as anxiety, depression, or PTSD emerge, persist, and interact. In turn, psychopathology can reinforce dysfunctional coping strategies, aggravating the severity of maladaptive traits over time. These aspects mutually reinforce each other: for instance, higher negative affectivity may lead to increased disinhibition, as individuals may engage in impulsive behaviors to cope with their distress; similarly, detachment and psychoticism may reduce engagement with social support, reinforcing affective and behavioral dysregulation.

These results highlight the complexity of vulnerability pathways in CVDs and the importance of going beyond reductionist models that focus on isolated traits or psychopathological disorders. This perspective is consistent with emerging frameworks, including the Hierarchical Taxonomy of Psychopathology (HiTOP; Kotov et al., 2017), the Psychodynamic Diagnostic Manual (PDM; Lingiardi and McWilliams, 2017), and research on transdiagnostic mechanisms. All these approaches underscore the need to consider interactions across multiple levels of psychological functioning rather than relying on categorical classifications alone. In this regard, ET—or rather, its disruptions—emerges as a relevant construct in the aftermath of MI, when patients are required to adopt lifestyle modifications and adhere to specific medical regimens. A collaborative relationship with healthcare providers can facilitate such efforts; however, since epistemic disruptions hinder the effective use and interpretation of interpersonally transmitted information, both EM and EC may represent a significant obstacle in the recovery process, hindering openness to professional advice and undermining patients' motivation to engage in health-promoting behaviors.

Within our sample, both EM and EC showed significant correlations with maladaptive personality traits and psychopathological symptoms. These findings align with literature suggesting that individuals who pervasively doubt or reject information conveyed through interpersonal exchanges may be more prone to cognitive distortions, impulsivity, and emotional dysregulation—showing a higher likelihood of developing psychopathological symptoms (Benzi et al., 2023; Campbell et al., 2021; Liotti et al., 2023). Furthermore, recent evidence indicated that high EM correlates with interpersonal difficulties and less adaptive coping strategies under stress (Fiorini Bincoletto et al., 2025), suggesting that mistrustful MI patients may struggle to benefit from social support or medical guidance precisely when such resources are most essential (e.g., Nimbi et al., 2023). EC might also expose patients to misinformation—especially in the age of internet-based health resources—leading them to follow unverified or counterproductive advice about their condition. However, while in our sample EC was also correlated with some maladaptive traits, these relationships were generally weaker or non-significant. Although credulity can expose individuals to questionable information, it may not necessarily hamper engagement with medical advice or psychosocial support to the same extent as EM. Epistemically credulous patients might still adhere adequately to evidence-based recommendations, buffering the effects of EC. Besides, the negative impact of EC may emerge prominently in contexts saturated with widespread misinformation or insufficient health literacy.

Since, as expected, the epistemic stance differentiating MI patients from controls was EM—which was also the epistemic variable most strongly correlated with both psychopathology and maladaptive personality traits—the study's third aim was to test whether EM might modulate the impact of personality on overall symptom severity (Table 3). Results showed that, although higher levels of personality impairment were associated with greater distress *per se*, this link was amplified among patients with elevated levels of EM. Such patients may doubt or dismiss guidelines, feedback, or psychoeducational input—particularly if these clash with their beliefs or emotional states. In the context of more severe personality impairment, mistrust might intensify maladaptive schemas and interpersonal patterns (e.g., suspicion, hostility, hyper-vigilance toward perceived criticism), as well as expose individuals to greater physiological and psychological reactivity to stress. Notably, the development of ET is thought to arise within secure attachment contexts, where infants learn to trust caregivers' cues and build reliable knowledge (Esposito et al., 2024). In contrast, insecure or disorganized attachment may foster epistemic disruptions (Fonagy and Allison, 2014). These attachment-related patterns, which show behavioral and neurophysiological stability, likely persist over time, disrupting ET and contributing to maladaptive interpersonal patterns (Chris Fraley, 2002; Cruciani et al., 2021; Lyons-Ruth et al., 2016; Paetzold et al., 2015; Zingaretti et al., 2020). Finally, EM can undermine the collaborative relationship with healthcare providers, leading MI patients to neither seek nor effectively implement adaptive strategies to cope, hindering the recovery processes. In this regard, it is important to acknowledge that in the present study patients were assessed before hospital discharge, which places them still in the acute phase of hospital stay; this could have influenced MI patients' psychological state, including levels of EM. In this vein, the emotional distress and uncertainty associated with hospitalization may have temporarily heightened mistrust, potentially inflating its association with psychopathology. However, this timeframe was chosen to ensure standardized assessment across participants and to capture the immediate psychological impact of MI. Importantly, even if disruptions in ET are transient, hospitalization and discharge periods are critical, as patients receive essential medical guidance on pharmacological treatments and lifestyle modifications. Thus, even temporary disruptions in ET can negatively impact patients' ability to process and integrate this information, directly affecting treatment adherence and long-term recovery outcomes. Future research would benefit from longitudinal designs to explore how EM evolves over time and whether it stabilizes or diminishes as patients adjust post-discharge.

### 4.1 Limitations and conclusions

This study has several limitations. MI sample was recruited from a single center in a Western country, limiting the generalizability of findings. Cultural variables may influence the relationship between psychopathological symptomatology, personality, and epistemic stances in MI patients, warranting further research. Additionally, despite the inclusion of a matched healthy control group (allowing to analyze distinctive psychological features of MI patients, thus representing a strength of the present study), the cross-sectional study design and the limited sample size pose another constraint for results generalizability and drawing causal inferences. Longitudinal studies are needed to address this issue. Similarly, the exclusive use of self-report measures may limit the generalizability of results due to possible participants' self-presentation biases, warranting future studies to adopt also implicit measures. A further limitation concerns the differences in clinical variables between MI patients and healthy controls. Given the exclusion criteria applied to the control group, individuals with major cardiovascular risk factors (e.g., obesity, smoking) were not included, leading to expected differences in these characteristics between groups. While this choice aimed to ensure a clear distinction and reduce the likelihood of including healthy individuals at risk for future MI, it also limits the generalizability of our findings. Future studies should consider exploring their potential moderating role in the relationship between epistemic trust, personality functioning, and psychopathological symptoms in MI patients. Similarly, future research is warranted to further address the potential role of other potentially significant factors, such as medical adherence and pre-existing psychiatric conditions, in affecting variables assessed in the present study. Nevertheless, this research aimed at focusing on subjectively reported mental health symptoms, in line with the patient-reported outcomes framework (Munyombwe et al., 2021; Norekvål et al., 2010; Savarese et al., 2023) and the scientific statement of the American Heart Association (Levine et al., 2021). Results suggest the need to promote a multidimensional assessment of MI patients' psychological functioning, as these variables play a pivotal role in shaping patients' recovery trajectories and long-term outcomes. Such assessment helps refine existing behavioral models of MI recovery, moving beyond anxiety-depression frameworks to encompass more nuanced constructs that shape how patients interpret and respond to medical advice. Specifically referring to ET, this construct may have significant implications in patients' capacity to engage with healthcare providers, as it may affect their ability to follow medical advice, adhere to treatment, and adopt protective behaviors like quitting smoking, improving diet, exercising, and managing stress—crucial for recovery. In fact, although EM originates from past experiences, it may also be amplified by the MI event, especially if patients feel overwhelmed, receive unclear medical information, or perceive a lack of support from healthcare providers. Poor communication or low empathy in the patient-provider relationship may further reinforce EM, reducing adherence to medical recommendations and promoting maladaptive coping behaviors. This perspective is crucial to developing targeted interventions to reduce barriers to care, improve patient engagement, and ultimately improve outcomes. In this vein, therapeutic interventions based on the triadic model proposed by Fonagy and colleagues could be effective in addressing epistemic stances, personality and psychological vulnerabilities among MI patients (Fisher et al., 2023; Fonagy and Campbell, 2017; Fonagy et al., 2015).

## Data Availability

The data that support the findings of this study are available from the authors upon reasonable request from the corresponding author.
