# Supplementary material for: Psychopathological symptoms, personality, and epistemic stances in individuals with myocardial infarction: an empirical investigation
Source: Front Psychol. 2025 May 22;16:1587747. doi: 10.3389/fpsyg.2025.1587747 (PMC12137302; doi:10.3389/fpsyg.2025.1587747)
Supplement: Supplementary file 1 [file Table_1.docx]

Supplementary Material

# Table S1

Descriptives and differences between MI patients and healthy controls on sociodemographic and clinical information.

|  | MI patients  (*n* = 67) | Healthy controls  (*n* = 80) |  |  |
| --- | --- | --- | --- | --- |
| *Sociodemographic information* | *M* (*SD*) | *M* (*SD*) | *t* (df) | *p* |
| Age (years) | 61.6 (9.77) | 59.0 (8.46) | 1.73 (145) | .086 |
|  | *n* (%) | *n* (%) | *Χ^2^*(df) | *p* |
| Gender  Females  Males | 16 (23.9)  51 (76.1) | 25 (31.3)  55 (68.7) | 0.99 (1) | .321 |
| Marital status  Unmarried  Married  Separated/divorced  Widower | 6 (9.0)  46 (68.7)  7 (10.4)  8 (11.9) | 12 (15.0)  59 (73.8)  6 (7.5)  3 (3.8) | 4.85 (3) | .183 |
| Educational level  Elementary school diploma  Middle school diploma  Higher school diploma  Bachelor’s or master’s degree  PhD or Specialization | 1 (1.5)  17 (25.4)  29 (43.3)  17 (25.4)  3 (4.5) | 1 (1.3)  8 (10.0)  37 (46.3)  29 (36.3)  5 (6.3) | 6.74 (4) | .150 |
| *Clinical information* | *M* (*SD*) | *M* (*SD*) | t(df) | *p* |
| BMI | 26.6 (4.00) | 24.0 (2.30) | 4.92 (145) | <.001 |
|  | *n* (%) | *n* (%) | *Χ^2^*(df) | *p* |
| BMI categories  Underweight  Normal weight  Overweight  Obesity class I  Obesity class II | 1 (1.5)  21 (31.3)  32 (47.8)  12 (17.9)  1 (1.5) | 2 (2.5)  52 (65.0)  26 (32.5)  0 (0)  0 (0) | 26.2 (4) | <.001 |
| Smoking  No  Yes, in the past  Yes, currently | 16 (23.9)  12 (17.9)  39 (58.2) | 49 (61.3)  31 (38.8)  0 (0) | 63.5 (2) | <.001 |
| Alcohol consumption  No  Yes, once or twice a week  Yes, more than twice a week | 34 (50.7)  5 (7.5)  28 (41.8) | 80 (100.0)  0 (0)  0 (0) | 50.8 (2) | <0.001 |
| Dyslipidaemia  No  Yes | 21 (31.3)  46 (68.7) | 74 (92.5)  6 (7.5) | 59.7 (1) | <.001 |
| Insulinemia  No  Yes | 57 (85.1)  10 (14.9) | 79 (98.8)  1 (1.3) | 9.9 (1) | .002 |
| Pulmonary oedema  No  Yes | 66 (98.5)  1 (1.5) | 80 (100.0)  0 (0) | 1.2 (1) | .273 |
| Transient ischemic attacks  No  Yes | 65 (97.0)  2 (3.0) | 80 (100.0)  0 (0) | 2.4 (1) | .120 |
| Renal failure  No  Yes | 63 (94.0)  4 (6.0) | 79 (98.8)  1 (1.3) | 2.5 (1) | .116 |
| Chronic obstructive pulmonary disease  No  Yes | 62 (92.5)  5 (7.5) | 80 (100.0)  0 (0) | 6.2 (1) | .013 |
| Sleep apnea  No  Yes | 65 (97.0)  2 (3.0) | 72 (90.0)  8 (10.0) | 2.8 (1) | .093 |
| Neoplasms  No  Yes | 64 (95.5)  3 (4.5) | 73 (91.3)  7 (8.8) | 1.1 (1) | .306 |
| Diabetes  No  Yes, type I  Yes, type II | 57 (85.1)  1 (1.5)  9 (13.4) | 79 (98.8)  1 (1.3)  0 (0) | 11.5 (2) | .003 |

*Note*. Percentages may not equal to 100, due to rounding. *n* = number of participants, *Χ^2^* = chi-square test statistic, *t* = Student’s t statistic, *df* = degrees of freedom, *p* = p value
